# Angiographic Evaluation and Endovascular Treatment Considerations of Brain Arteriovenous Malformations With a Transdural Blood Supply: A Single-Center Experience

**DOI:** 10.3389/fneur.2020.603256

**Published:** 2021-01-20

**Authors:** Kun Hou, Kan Xu, Lai Qu, Guichen Li, Yunbao Guo, Jinlu Yu

**Affiliations:** ^1^Department of Neurosurgery, The First Hospital of Jilin University, Changchun, China; ^2^Department of Intensive Care Unit, The First Hospital of Jilin University, Changchun, China; ^3^Department of Neurology, The First Hospital of Jilin University, Changchun, China

**Keywords:** brain arteriovenous malformation, endovascular treatment, endovascular embolization, transdural blood supply, middle meningeal artery

## Abstract

**Background:** In rare circumstances, brain arteriovenous malformations (BAVMs) can recruit a transdural blood supply (TBS). The clinical and radiologic characteristics of BAVMs with a TBS are poorly understood.

**Methods:** A retrospective review of the medical records was conducted for adult patients who were admitted for BAVMs from Jan 2013 to Dec 2019. TBSs for BAVMs were divided into 3 types: (1) unilateral TBSs from the external carotid artery (ECA) and/or meningeal branch of the vertebral artery (VA); (2) bilateral TBSs from the ECA and/or meningeal branch of the VA; and (3) meningohypophyseal trunk TBSs of the internal carotid artery.

**Results:** Four hundred and twenty-eight patients were diagnosed with BAVMs during the study period, of whom 30 (7.0%, 30/428) were identified as having a TBS. Type 1, type 2, and type 3 TBSs were identified in 21 (70%, 21/30), 7 (23.3%, 7/30), and 2 (6.7%, 2/30) patients, respectively. Six (20%, 6/30) patients were conservatively managed. Twelve (40%, 12/30) patients underwent endovascular treatment (EVT) of the BAVM through non-TBS feeders. Eight (26.8%, 8/30) patients underwent EVT of the BAVM both through the TBS and non-TBS feeders. The modified Rankin Scale scores at the 3-month follow-up were 0, 1, 2, 4, and 5 in 24 (80%, 24/30), 2 (6.7%, 2/30), 2 (6.7%, 2/30), 1 (3.3%, 1/30), and 1 (3.3%, 1/30) patients, respectively. Good short-term recovery was achieved in 86.7% (26/30) of the patients. The size of the BAVMs with a TBS was larger than that of BAVMs without a TBS. Patients with higher Spetzler-Martin grades tended to have a TBS. No statistical difference was noted between the patients with and without a TBS with regard to age, sex, location, or concurrent aneurysms.

**Conclusions:** This study showed that a TBS was likely to develop in patients with larger BAVMs and that a TBS was likely to be located in the temporal lobe in patients BAVMs with higher SM grades. Weak structures were the primary targets of management. In addition, a BAVM could be embolized via the TBS.

## Introduction

Brain arteriovenous malformation (BAVM) is an abnormal nidus lacking a capillary bed between the cerebral arteries and veins. In general, a BAVM receives its blood supply from the subdural cerebral arteries. However, in rare circumstances, a BAVM can recruit a transdural blood supply (TBS), which is more common in dural arteriovenous fistula (DAVF) and moyamoya disease (MMD) (1). A TBS may be derived from meningeal branches of the internal carotid artery (ICA), the external carotid artery (ECA), and the vertebral artery (VA) (2).

According to past reports, a BAVM with a TBS is uncommon, the incidence of which ranges from 6 to 7% (1–4). Therefore, the clinical and radiologic characteristics of BAVMs with a TBS are poorly understood. Currently, studies focusing on BAVMs with a TBS are scarce, and only a few reports have been published (1, 3, 5). Due to the rarity of this entity, the published studies only had small sample sizes. The information that we can obtain from these studies is limited. In this study, we present a single-center experience on the clinical and radiological characteristics of BAVMs with a TBS.

In general, the management of a BAVM includes conservative and interventional treatments. Interventional treatments comprise endovascular treatment (EVT), microsurgery, and stereotactic radiosurgery. As a minimally invasive option, EVT can stand as a curative treatment or an adjunctive treatment to microsurgery and/or stereotactic radiosurgery. EVT has become an increasingly preferred option (6). In this study, we also provide our understanding of EVT for BAVMs with a TBS.

## Methods and Materials

A retrospective review of the medical records was conducted for adult patients who were admitted for a BAVM from Jan 2013 to Dec 2019. The patients were recruited consecutively. This study was approved by the Ethics Committee of The First Hospital of Jilin University. The inclusion criteria were as follows: (a) patients must have 6-vessel and super-selective angiogram, (b) with a TBS, and (c) no previous treatment (endovascular or surgical) for a BAVM and/or a TBS. The radiological investigations were independently evaluated by three senior neurointerventional specialists (Kun Hou, Kan Xu, and Jinlu Yu). In cases of disagreement among the evaluators, an additional specialist was consulted.

### BAVM Angioarchitecture

The location and feeding artery of each BAVM and flow-related aneurysm in the feeding artery were recorded. The nidus of each BAVM was divided into compact and diffuse types. The draining characteristics, including deep venous drainage and retrograde flow, were also recorded.

### TBS Classification

**Figure 1:**
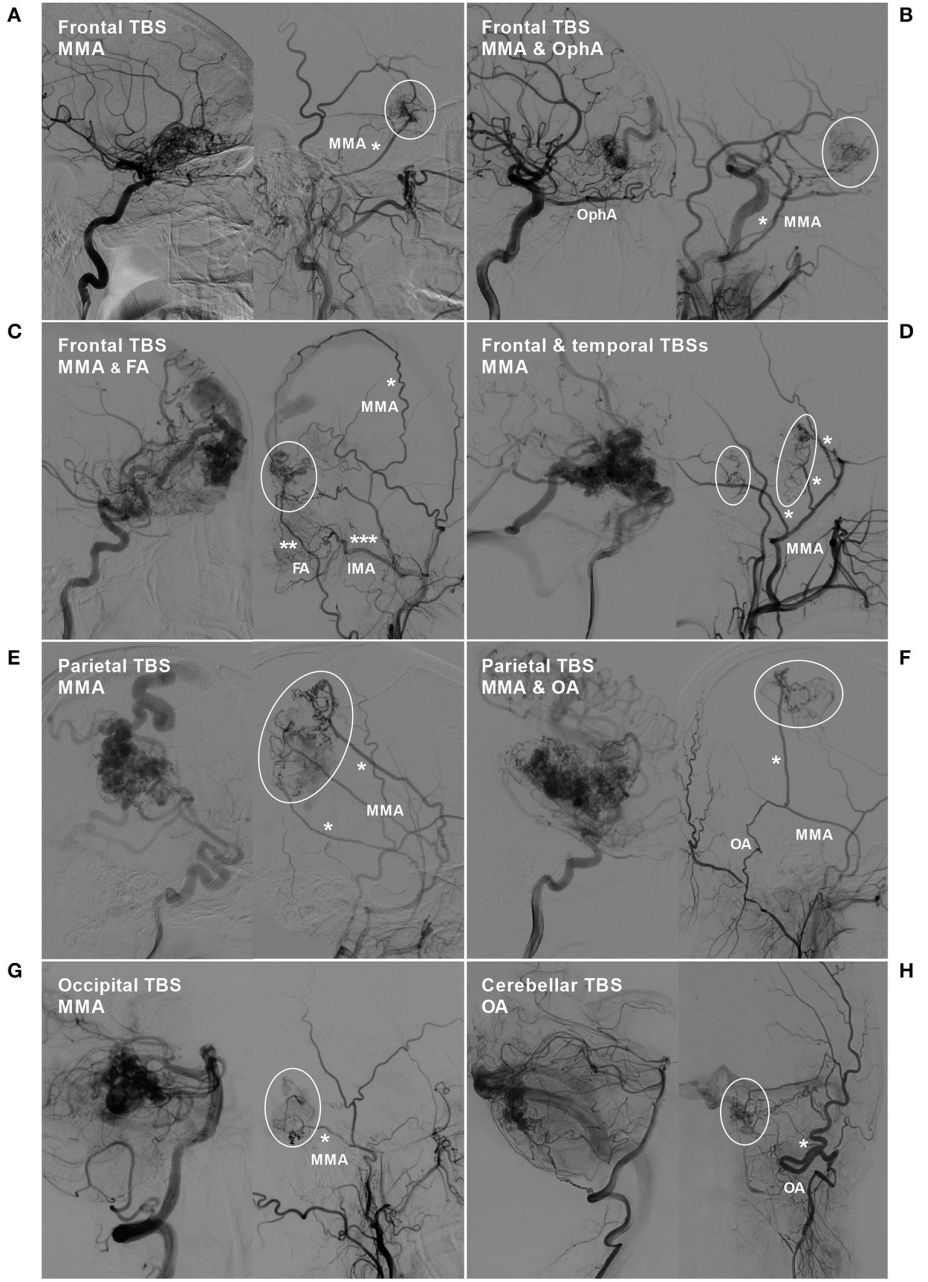
BAVM with a unilateral TBS. **(A)** Angiogram of the left ICA (left) and ECA (right) in lateral view shows a BAVM in the frontal lobe, which also receives TBS (encircled area) from the MMA (asterisk). **(B)** Angiogram of the left ICA (left) and ECA (right) in lateral view shows a frontal BAVM, which also receives TBS (encircled area) from the ipsilateral OphA and MMA (asterisk). **(C)** Angiogram of the ICA (left) and ECA (right) reveals a frontal BAVM, which also receives TBS (encircled area) from the MMA (single asterisk), FA (double asterisks), and IMA (triple asterisks). **(D)** Angiogram of the ICA (left) and ECA (right) reveals a frontotemporal BAVM, which also receives TBS (encircled area) from multiple branches (asterisks) of the MMA. **(E)** Angiogram of the ICA (left) and ECA (right) reveals a frontal BAVM, which also receives TBS (encircled area) from multiple branches (asterisks) of the MMA. **(F)** Angiogram of the ICA (left) reveals a parietal BAVM. Angiogram of the ECA (right) shows that the meningeal branch of the OA and the MMA converge (asterisk) to provide TBS (encircled area) to the BAVM. **(G)** Angiogram of the VA (left) and ECA (right) reveals an occipital BAVM, which also receives TBS (encircled area) from the MMA (asterisk). **(H)** Angiogram of the VA (left) and ECA (right) reveals a cerebellar BAVM, which also receives TBS (encircled area) from the meningeal branch (asterisk) of the OA. BAVM, brain arteriovenous malformation; ECA, external carotid artery; FA, facial artery; ICA, internal carotid artery; IMA, internal maxillary artery; MMA, middle meningeal artery; OA, occipital artery; OphA, ophthalmic artery; TBS, transdural blood supply; VA, vertebral artery.

**Figure 2:**
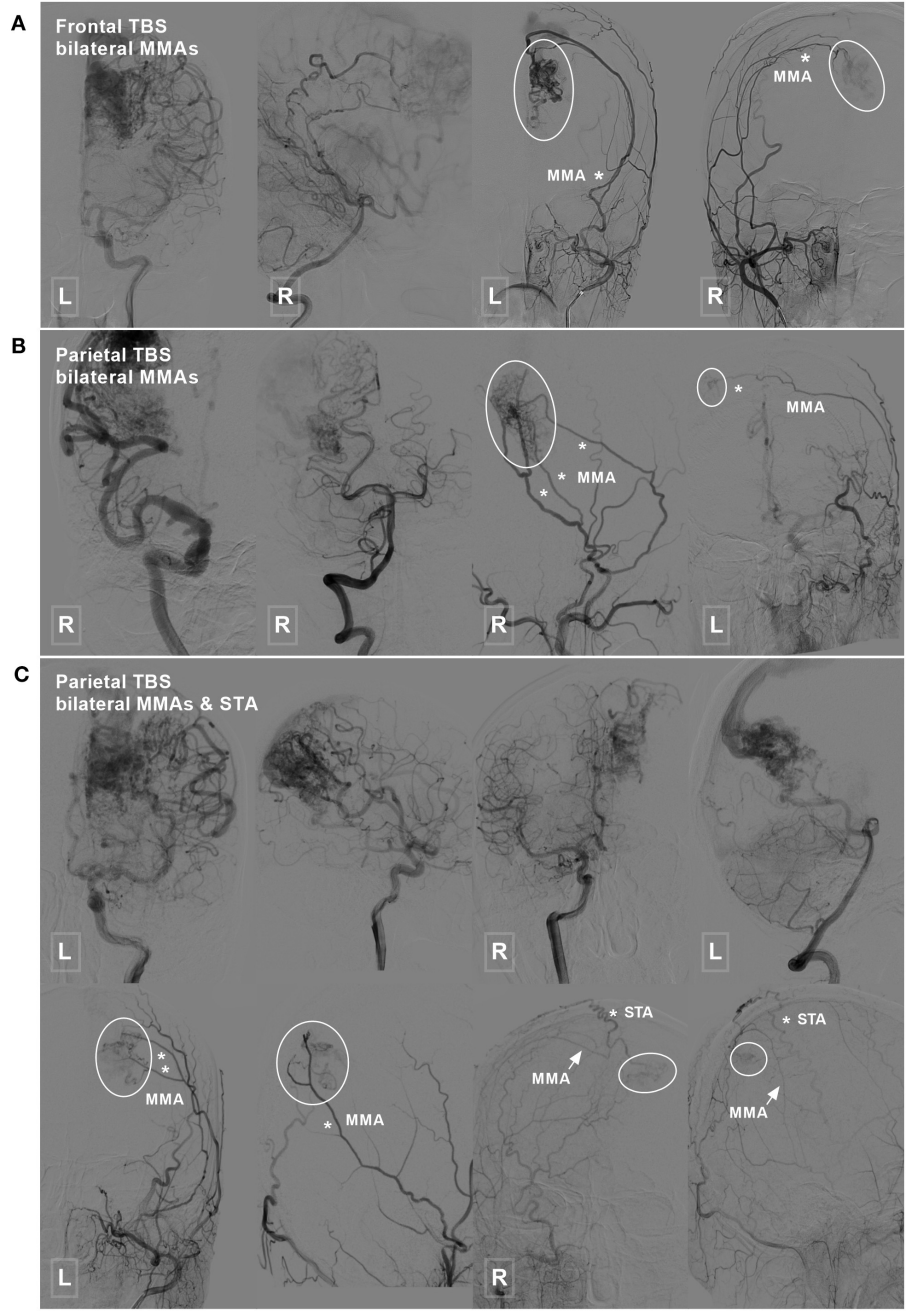
Frontal and parietal BAVM with a bilateral TBS. **(A)** Angiogram of the ICA (left 1) and VA (left 2) reveals a frontal BAVM. Angiogram of the bilateral ECA (right 1 and 2) shows that this BAVM also receives TBS (encircled area) from the bilateral MMA. **(B)** Angiogram of the ICA (left 1) and VA (left 2) reveals a parietal BAVM. Angiogram of the bilateral ECA (right 1 and 2) shows that the bilateral MMA (asterisk) also provide TBS (encircled area) to this BAVM. **(C)** Angiogram of the ICA (upper row left 1, 2, and right 1) and VA (upper row right 2) reveals a parietal BAVM. Angiogram of the bilateral ECA (lower row) shows the bilateral MMA (asterisk in left 1 and 2, arrow in right 1 and 2), and the right STA (asterisk in right 1 and 2) also provides TBS (encircled area) to the BAVM. BAVM, brain arteriovenous malformation; ECA, external carotid artery; ICA, internal carotid artery; MMA, middle meningeal artery; STA, superficial temporal artery; TBS, transdural blood supply; VA, vertebral artery.

**Figure 3:**
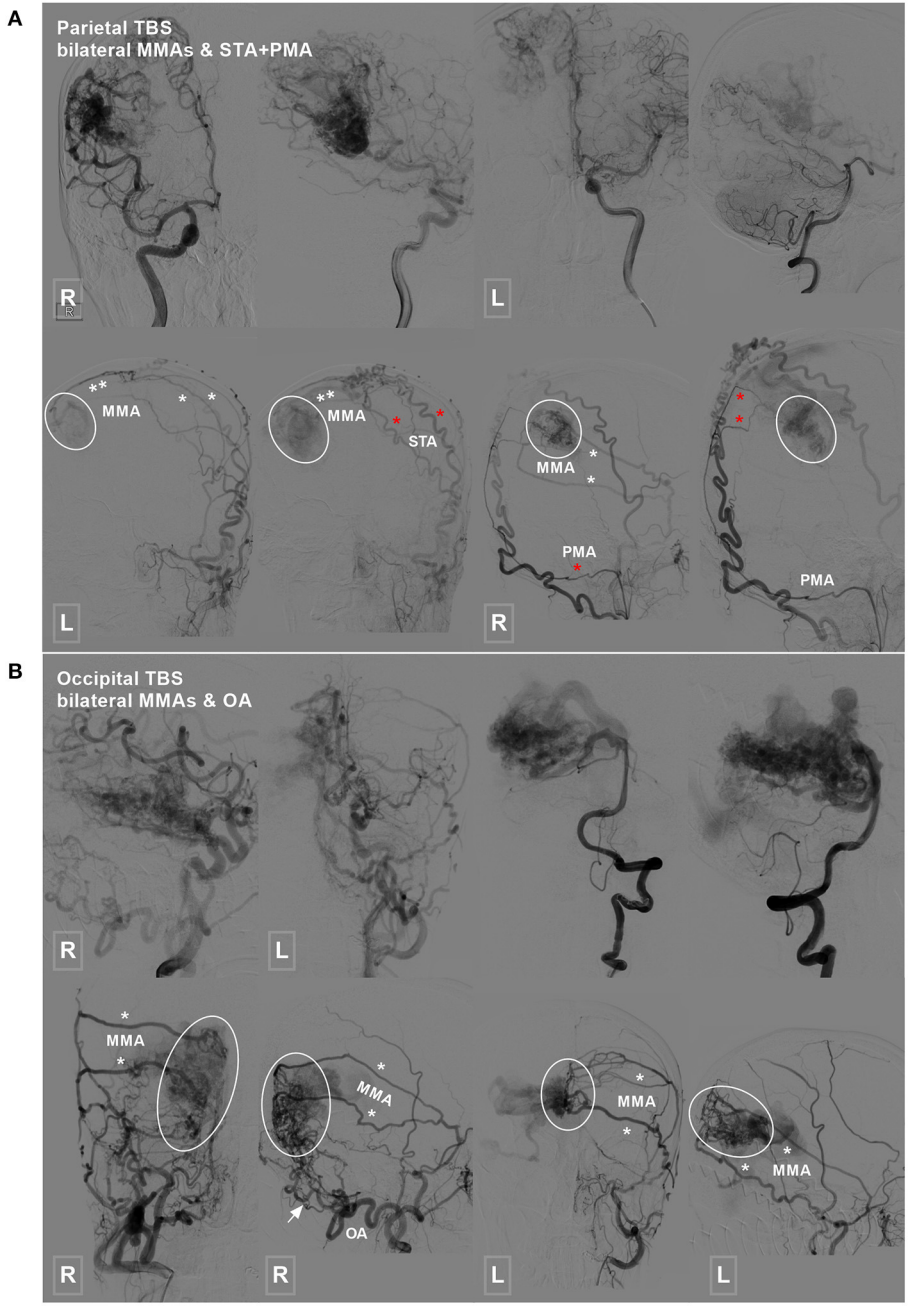
Parietal and occipital BAVM with a bilateral TBS. **(A)** Angiogram of the ICA (upper row left 1, 2, and right 1) and VA (upper row right 2) reveals a parietal BAVM. Angiogram of the bilateral ECA (lower row) shows that the bilateral MMA (white asterisk), left STA (red asterisk), and right PMA (red asterisk) also provide TBS (encircled area) to the BAVM. **(B)** Angiogram of the ICA (upper row left 1 and 2) and VA (upper row right 1 and 2) reveals an occipital BAVM. Angiogram of the bilateral ECA (lower row) shows that the BAVM also receives TBS (encircled area) from the bilateral MMA (asterisk) and right OA (arrow). BAVM, brain arteriovenous malformation; ECA, external carotid artery; ICA, internal carotid artery; MMA, middle meningeal artery; OA, occipital artery; PMA, posterior meningeal artery; STA, superficial temporal artery; TBS, transdural blood supply; VA, vertebral artery.

**Figure 4:**
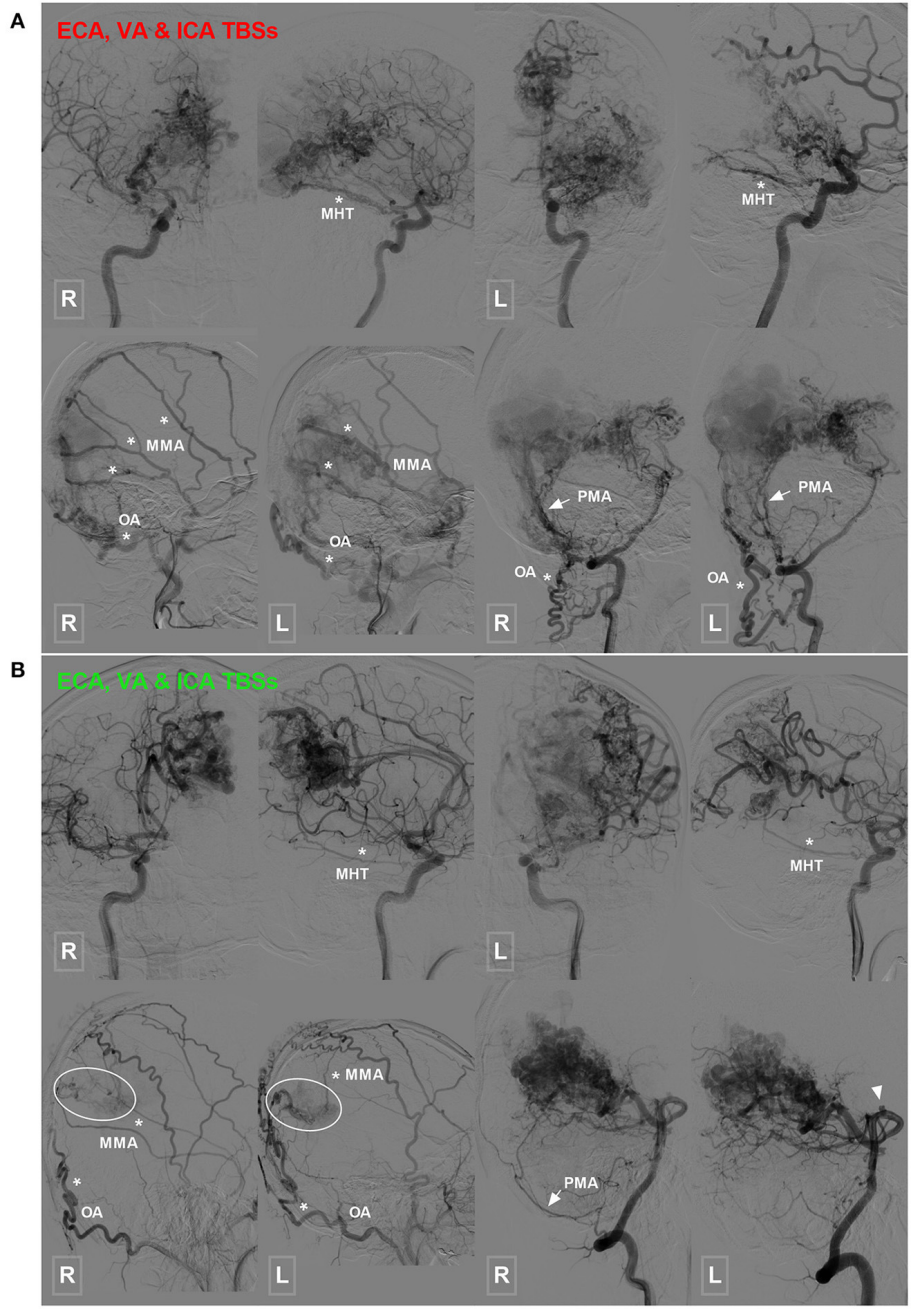
Occipital BAVM with a TBS from the MHT. **(A)** Angiogram of the bilateral ICA (upper row) reveals an occipital BAVM that receives TBS from the bilateral MHT (asterisk). Angiogram of the bilateral ECA (lower row left 1 and 2) and VA (lower row right 1 and 2) shows that the bilateral MMA (asterisk), OA (asterisk), and PMA (arrow) also provide TBS to the BAVM. A muscle branch of the OA anastomoses with the VA. **(B)** Angiogram of the bilateral ICA (upper row) reveals an occipital BAVM that receives TBS from the bilateral MHT (asterisk). Angiogram of the bilateral ECA (lower row left 1 and 2) and VA (lower row right 1 and 2) shows that the bilateral MMA (asterisk) and OA (asterisk), and right PMA (arrow) also provide TBS (encircled area) to the BAVM. The bilateral PCA (right 1 and 2) also supply the BAVM, and a flow-related aneurysm (arrowhead) is noted at the proximal segment of the PCA. BAVM, brain arteriovenous malformation; ECA, external carotid artery; ICA, internal carotid artery; MHT, meningohypophyseal trunk; MMA, middle meningeal artery; OA, occipital artery; PCA, posterior cerebral artery; PMA, posterior meningeal artery; TBS, transdural blood supply; VA, vertebral artery.

TBSs to the BAVMs were divided into the following types: (1) unilateral TBS from the ECA and/or meningeal branch of the VA (Figure 1); (2) bilateral TBS from the ECA and/or meningeal branch of the VA (Figures 2, 3); and (3) meningohypophyseal trunk (MHT) TBS of the ICA (Figure 4).

### EVT Strategy for a BAVM With a TBS

The primary aim of EVT for BAVMs was targeted management of ruptured or weak structures (7). The ruptured or weak structures included hemodynamic aneurysms on the feeding artery and intranidal aneurysms.

#### The EVT for Aneurysms

When a flow-related aneurysm was far away from the BAVM nidus, coiling, or stent-assisted coiling was performed. When the flow-related aneurysm was close to the BAVM nidus, parent artery occlusion (PAO) of the feeding artery was preferred.

#### The EVT for BAVMs

For ruptured BAVMs, EVT of the weak points were the primary target. If no weak point was identified, partial or curative embolization of the BAVM to decrease the blood volume was performed. For unruptured BAVMs, weak points were the main target of treatment. If no weak point was identified, a wait-and-see strategy was adopted.

#### EVT for TBSs

For enlarged TBS arteries with high blood volume, EVT of the TBS arteries with Onyx was performed if needed to embolize the BAVM nidus. If the TBS arteries were small, no further treatment was needed.

### Follow-Up

The patients were followed up in the outpatient department or by telephone investigation. The outcome was evaluated with the modified Rankin Scale (mRS) (8). mRS scores of 0 and 1 were defined as good recovery.

### Statistical Analysis

Statistical assessment was performed using SPSS 25.0 (IBM Corp., Armonk, NY, USA). Continuous variables were expressed as the mean ± standard deviation, and differences were assessed with a *t*-test. The chi-square test or Fisher's exact test was used to analyze count data. *P* < 0.05 was considered statistically significant.

## Results

### General Information

Four hundred twenty-eight patients were diagnosed with BAVMs during the study period, of whom thirty (7.0%, 30/428) were shown to have a TBS. The 30 patients with a TBS were aged from 22 to 62 (39.4 ± 12.4) years. The male to female ratio was 2:1. The clinical manifestations were headache, dizziness, seizure, and intracranial hemorrhage in 20 (66.7%, 20/30), 1 (3.3%, 1/30), 2 (6.7%, 2/30), and 7 (23.3%, 7/30) patients, respectively. Of the 7 patients presenting with intracranial hemorrhage, 3 presented with intracerebral hemorrhage, 2 presented with intraventricular hemorrhage, 1 presented with intracerebral hemorrhage with ventricular involvement, and 1 presented with subarachnoid hemorrhage.

### Angiographic Characteristics

#### BAVM Nidus

**Figure 5:**
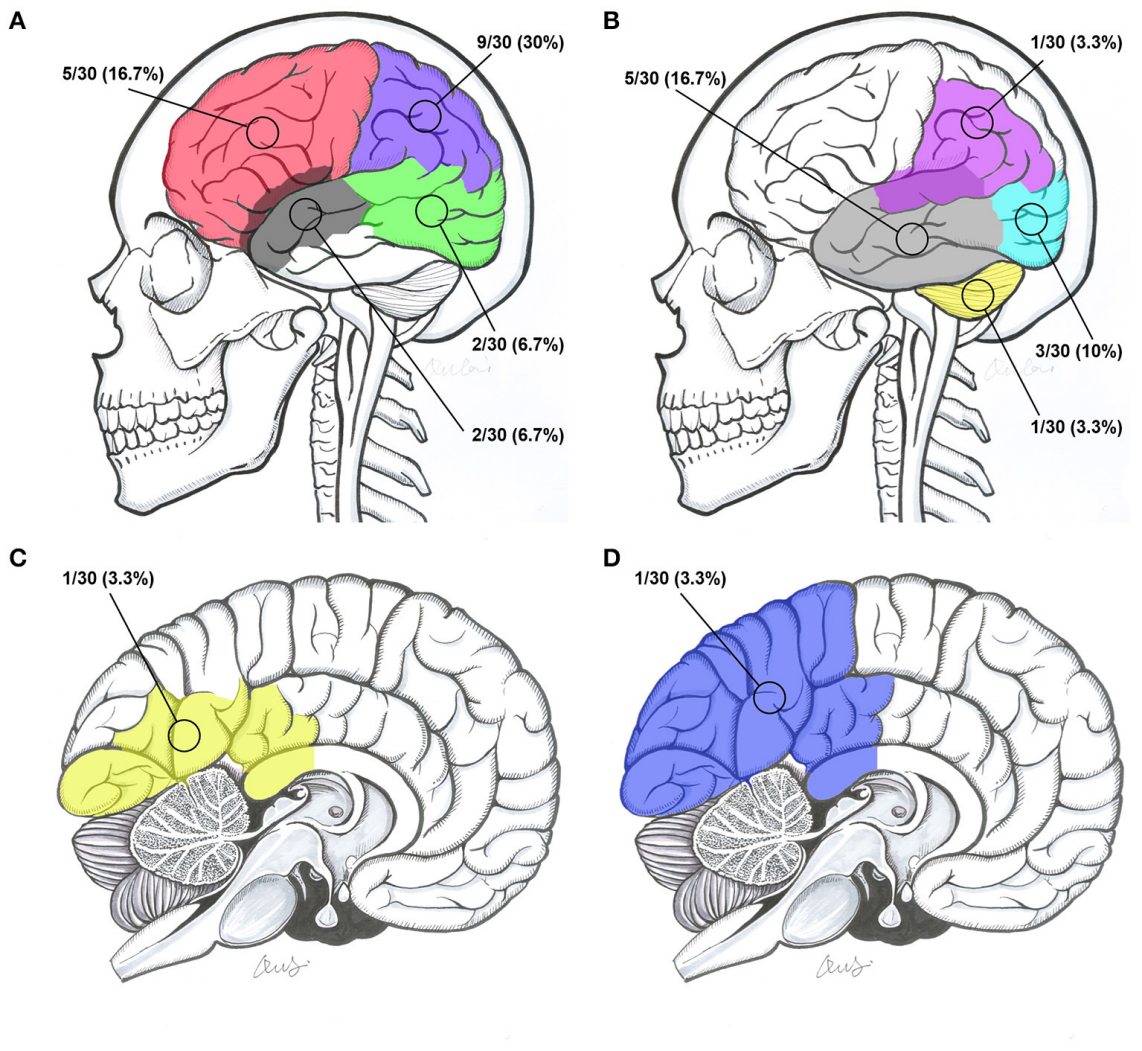
Intracranial locations of BAVMs. **(A)** BAVMs located in the frontal lobe (red), parietal lobe (purple), occipitotemporal lobe (green), and temporal lobe involving the sylvian fissure (gray). **(B)** BAVMs located in the temporal lobe (gray), occipital lobe (indigo), cerebellum (yellow), and parietal lobe involving the sylvian fissure (purple). **(C)** BAVMs located in the occipital lobe involving the callosum (yellow). **(D)** A BAVM located at the parietooccipital lobe involving the callosum (blue). BAVM, brain arteriovenous malformation.

The locations of the BAVM nidi were the frontal lobe, parietal lobe, temporal lobe, occipital lobe, occipitotemporal lobe, temporal lobe involving the sylvian fissure, parietal lobe involving the sylvian fissure, parietooccipital lobe involving the callosum, occipital lobe involving the callosum, and cerebellum in 5 (16.7%, 5/30), 9 (30%, 9/30), 5 (16.7%, 5/30), 3 (10%, 3/30), 2 (6.7%, 2/30), 2 (6.7%, 2/30), 1 (3.3%, 1/30), 1 (3.3%, 1/30), 1 (3.3%, 1/30), and 1 (3.3%, 1/30) patients, respectively (Figure 5).

The BAVM sizes ranged from 2 to 8.5 (5.2 ± 1.8) cm. Regarding the classifications of the BAVM nidi, 22 (73.3%, 22/30) were of the compact type, and 8 (26.7%, 8/30) were of the diffuse type.

#### Feeding Artery of the BAVMs

The BAVMs received their blood supply from the anterior circulation, posterior circulation, and both the anterior and posterior circulation in 9 (30%, 9/30), 4 (13.3%, 4/30), and 17 (56.7%, 17/30) patients, respectively. Five (16.7%, 5/30) patients were identified as having flow-related aneurysms.

#### Draining the Veins of the BAVMs

Twenty-one (70%, 21/30) patients had only superficial venous drainage. The remaining 9 (30%, 9/30) patients had both superficial and deep venous drainage. Of the 9 patients with combined superficial and deep venous drainage, 6 were mainly drained by superficial veins, and 3 were mainly drained by deep veins. Retrograde flow in the ipsilateral or contralateral venous sinuses was identified in 8 (26.7%, 8/30) patients.

#### Spetzler-Martin Grading

The Spetzler-Martin (SM) grades were 1, 2, 3, 4, and 5 in 2 (6.7%, 2/30), 6 (20%, 6/30), 7 (23.3%, 7/30), 12 (40%, 12/30), and 3 (10%, 3/30) patients, respectively.

#### TBS Angioarchitecture

Type 1: A unilateral TBS from the ECA and/or meningeal branch of the VA was identified in 21 (70%, 21/30) patients. The feeding TBSs were from the middle meningeal artery (MMA), occipital artery (OA), MMA + superficial temporal artery (STA), MMA + OA, MMA + ophthalmic artery (OphA), and MMA + facial artery (FA) + internal maxillary artery (IMA) in 14 (66.7%, 14/21), 2 (9.5%, 2/21), 2 (9.5%, 2/21), 1 (4.8%, 1/21), 1 (4.8%, 1/21), and 1 (4.8%, 1/21) patients, respectively.

Type 2: Bilateral TBS from the ECA and/or meningeal branch of the VA was identified in 7 (23.3%, 7/30) patients. The feeding TBSs were from the bilateral MMA, bilateral MMA + bilateral OA, bilateral MMA + unilateral OA, bilateral MMA + unilateral STA + unilateral posterior meningeal artery (PMA), and bilateral MMA + unilateral STA in 3 (42.8%, 3/7), 1 (14.3%, 1/7), 1 (14.3%, 1/7), 1 (14.3%, 1/7), and 1 (14.3%, 1/7) patients, respectively.

Type 3: A TBS with MHT participation was identified in 2 (6.7%, 2/30) patients, of whom 1 exhibited participation from the bilateral MHT, OA, MMA, and PMA and the other exhibited participation from the bilateral OA, MMA, MHT, and unilateral PMA.

The TBS was enlarged in 13 (43.3%, 13/30) patients. The arteries providing the TBS to the BAVM and their respective frequencies of occurrence are presented in Table 1.

**Table 1:** TBS origins and frequencies of occurrence.

**Origin**	**Frequency of occurrence**
MMA	93.3% (28/30)
OA	23.3% (7/30)
STA	13.3% (4/30)
PMA	10% (3/30)
MHT	6.7% (2/30)
IMA	3.3% (1/30)
FA	3.3% (1/30)
OphA	3.3% (1/30)

*BAVM, brain arteriovenous malformation; FA, facial artery; IMA, internal maxillary artery; MHT, meningohypophyseal trunk; MMA, middle meningeal artery; OA, occipital artery; OphA, ophthalmic artery; PMA, posterior meningeal artery; STA, superficial temporal artery; TBS, transdural blood supply*.

#### Statistical Analysis

The sizes of BAVMs with a TBS were larger than those of BAVMs without a TBS. Patients with higher SM grades tended to have a TBS. No statistical difference was noted between the patients with and without a TBS regarding age, sex, location, or concurrent aneurysms. The results of the statistical analysis are presented in Table 2.

**Table 2:** Statistical analysis of the patients with and without a TBS.

	**With a TBS**	**Without a TBS**	***P* value**
Age (mean ± SD)	39.4 ± 12.4 years	35.3 ± 16.8 years	0.1993
F (%)	10 (10/30, 33.3%)	188 (188/398, 47.2%)	0.0568
Subtentorial location (%)	1 (1/30, 3.3%)	63 (63/398, 15.8%)	0.0660
Concurrent with aneurysms (%)	5 (5/30, 16.7%)	72 (72/398, 18.1%)	>0.9999
Size (mean ± SD)	5.2 ± 1.8 cm	4.0 ± 2.2 cm	0.0046
SM (I+II grade) (%)	8 (8/30, 26.7%)	243 (243/398, 61.1%)	0.0004

*F, female; SD, standard deviation; SM, Spetzler-Martin; TBS, transdural blood supply*.

### EVT Options

**Figure 6:**
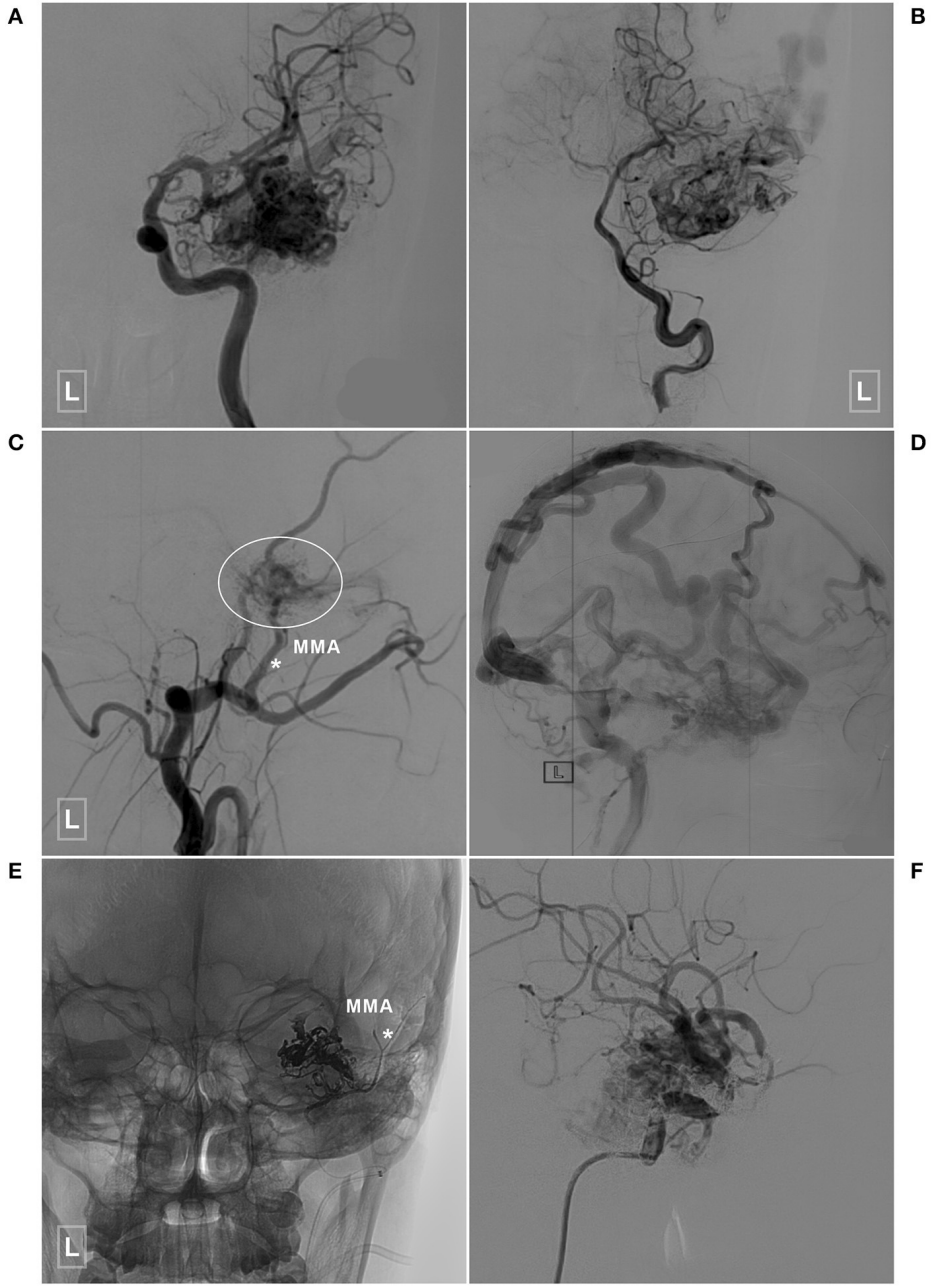
An illustrative case of a BAVM with a TBS undergoing embolization of the TBS and BAVM nidus. **(A,B)** Angiogram of the left ICA **(A)** and VA **(B)** reveals a temporal BAVM supplied by both the ICA and PCA. **(C)** Angiogram of the left ECA in the lateral view shows that the BAVM (encircled area) also receives TBS from the MMA (asterisk). **(D)** Angiogram of the left ICA in the lateral view at the venous phase reveals extensive venous drainage. **(E)** X-ray of the cranium shows the TBS from the MMA (asterisk), and the BAVM has been embolized with Onyx. **(F)** Selective angiogram of the left ICA shows embolization of the BAVM. BAVM, brain arteriovenous malformation; ICA, internal carotid artery; MMA, middle meningeal artery; PCA, posterior cerebral artery; TBS, transdural blood supply; VA, vertebral artery.

**Figure 7:**
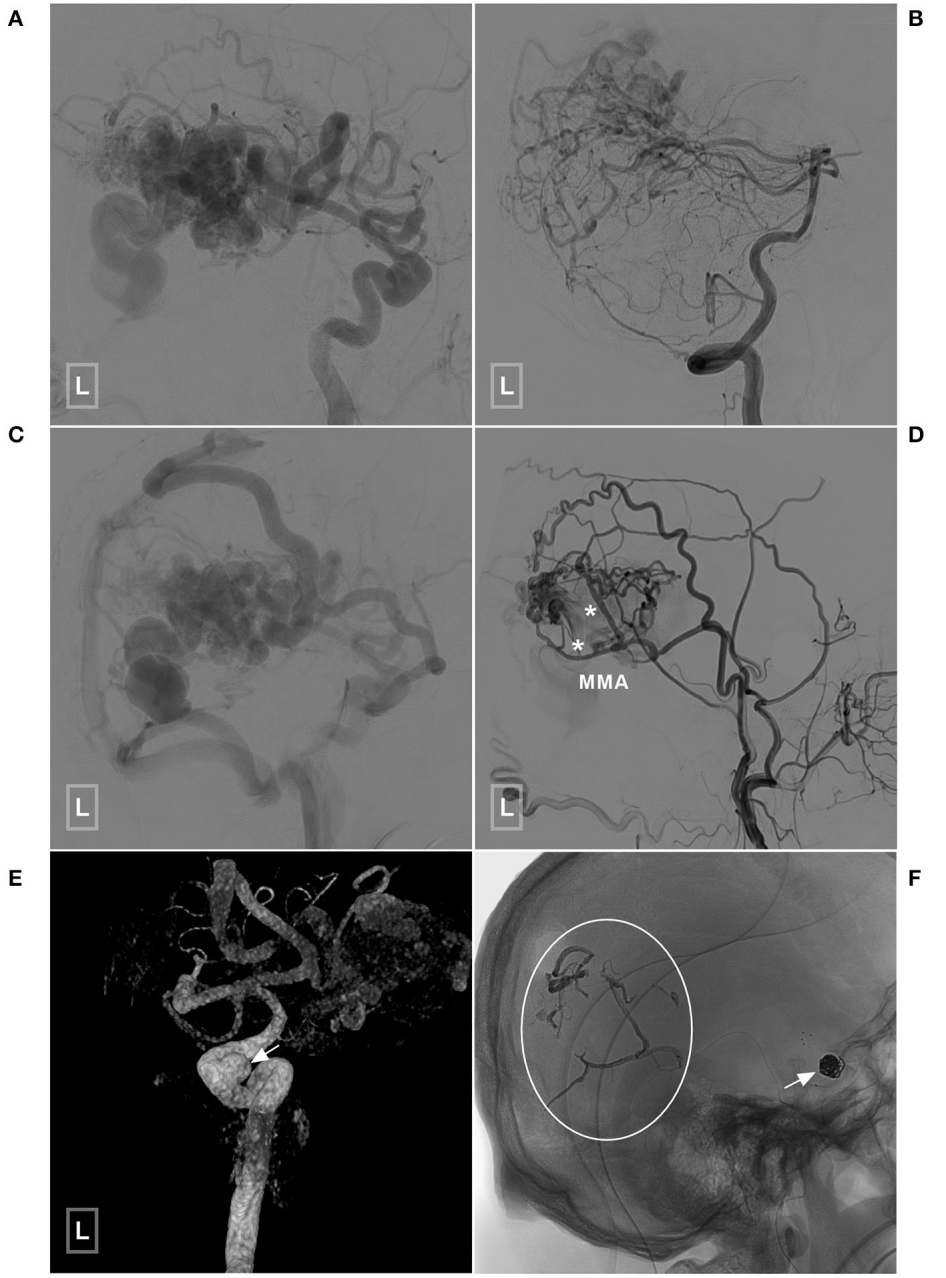
An illustrative case of BAVM with a TBS undergoing embolization of the flow-related aneurysm and TBS. **(A,B)** Angiogram of the left ICA **(A)** and VA **(B)** reveals a temporal BAVM supplied by both the ICA and PCA. **(C)** Angiogram of the left ICA in the venous phase shows that most of the veins of the left hemisphere are involved. **(D)** Angiogram of the left ECA shows that the BAVM also receives TBS from the MMA (asterisks). **(E)** Three-dimensional angiogram reveals an aneurysm (arrow) at the cavernous segment of the ICA. **(F)** The aneurysm has been coiled with stent assistance (arrow). The TBS has been embolized (encircled area). BAVM, brain arteriovenous malformation; ECA, external carotid artery; ICA, internal carotid artery; MMA, middle meningeal artery; PCA, posterior cerebral artery; TBS, transdural blood supply; VA, vertebral artery.

Six (20%, 6/30) patients were conservatively managed. Twelve (40%, 12/30) patients underwent EVT of the BAVM through non-TBS feeders. Eight (26.8%, 8/30) patients underwent EVT of the BAVM both through the TBS and non-TBS feeders. One (3.3%, 1/30) patient underwent coiling of the flow-related aneurysm while leaving the BAVM intact. Three (10%, 3/30) patients underwent embolization of both the aneurysm and the BAVM. Two illustrative cases are presented in Figures 6, 7.

### Follow-Up and Outcome

**Figure 8:**
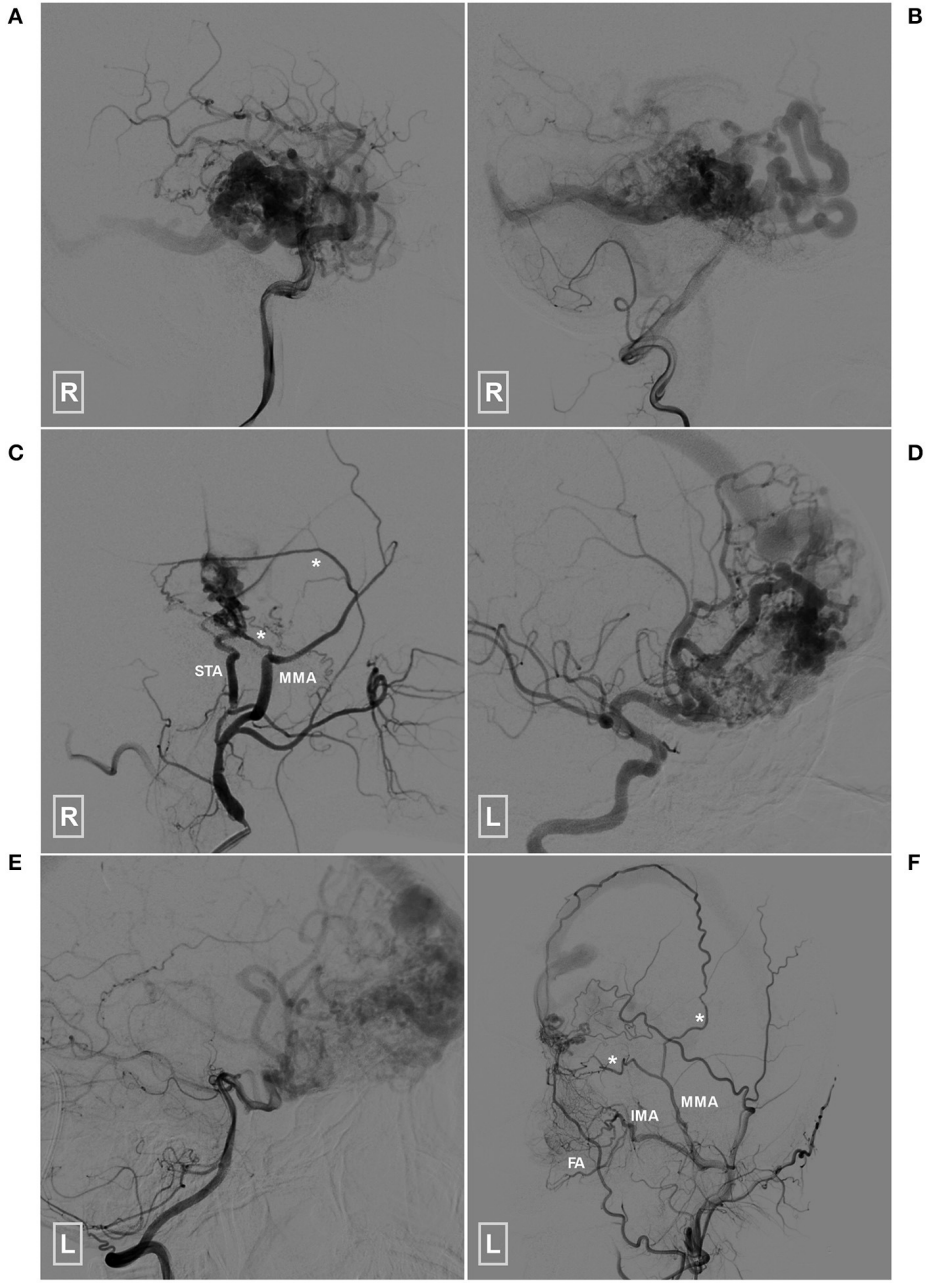
Angioarchitecture of the two patients who died during the follow-up period **(A–C)** (patient 1), Angiogram of the right ICA **(A)** and VA **(B)** reveals a temporal BAVM supplied by both the ICA and PCA. **(C)** Angiogram of the right ECA shows that the BAVM also receives TBS from the MMA (asterisks) and STA. **(D–F)** (patient 2), Angiogram of the left ICA **(D)** and VA **(E)** reveals a temporal BAVM supplied by both the ICA and PCA. **(F)** Angiogram of the left ECA shows that the BAVM also receives TBS from the MMA (asterisks), FA and IMA. BAVM, brain arteriovenous malformation; ECA, external carotid artery; FA, facial artery; ICA, internal carotid artery; IMA, internal maxillary artery; MMA, middle meningeal artery; PCA, posterior cerebral artery, STA, superficial temporal artery; TBS, transdural blood supply; VA, vertebral artery.

The follow-up period ranged from 3 to 60 (26.1 ± 20.4) months. The mRS scores at the 3-month follow-up were 0, 1, 2, 4, and 5 in 24 (80%, 24/30), 2 (6.7%, 2/30), 2 (6.7%, 2/30), 1 (3.3%, 1/30), and 1 (3.3%, 1/30) patients, respectively. Good short-term recovery (mRS of 0 and 1) was achieved in 86.7% (26/30) of the patients. Two (6.7%, 2/30) patients died due to rebleeding during the follow-up period; of these two patients, 1 died 1 year and 8 months after discharge and the other died 2 years and 11 months after discharge. The angiograms of the two patients who died are presented in Figure 8. The remaining 28 patients still participated in follow-up evaluations and reported no remarkable events.

## Discussion

For some cerebrovascular diseases, such as MMD and DAVF, a TBS is common, and the MMA is the most common source of a TBS (9, 10). In addition, other branches of the ECA and meningeal branches of the ICA and VA can also provide TBS (9, 11, 12). The main feeding arteries of a BAVM are mostly the ICA and VA. In rare circumstances, the BAVM can also recruit a TBS from the ECA or meningeal branches of the ICA and VA (2). A TBS to a BAVM is rare, the actual incidence of which remains to be elucidated. According to Koo and Bervini et al.'s studies, the incidence was reported to be ~7% (3, 4). In our study, the incidence was 7%, which is similar to past reports.

### Angioarchitecture of a TBS to a BAVM

A TBS to a BAVM has similar angioarchitecture with that of MMD and DAVF. In this study, a TBS from the MMA was identified in 93.3% of the patients, followed by OA, which was identified in 23.3% of the patients. A TBS from the PMA, originating from the ECA or VA, was identified in 10% of the patients (Table 1). Other branches of the ECA (e.g., the STA, FA, and IMA) and the ICA (e.g., the ethmoid artery from the OphA and MHT) were also identified to provide TBS, which indicated that both the extra and intracranial sources of meningeal branches can provide TBS to a BAVM. Under normal conditions, the meningeal branches of the extra and intracranial arteries are small and angiographically invisible. However, when providing a TBS to the BAVM, these branches often become enlarged due to the increased hemodynamic stress. In our study, 43.3% of the transdural arteries were enlarged.

### Pathogenesis of TBS in BAVM

The cause of a TBS poses an interesting question. To date, the precise pathophysiological mechanism of a TBS is still obscure. A TBS may develop simultaneously with the genesis of BAVMs or develop after the formation of BAVMs (2, 4). According to Bervini et al., there are 3 possible explanations for the genesis of a TBS to a BAVM, which include (1) the development of a TBS upon the generation of the BAVM; (2) recruitment of transdural collaterals to the BAVM as a result of the BAVM-induced hypoxic environment; and (3) recruitment of the transdural arteries as a result of the BAVM-induced high wall shear stress (4). However, no consensus on the mechanism has been established until now.

There are as least 3 issues that need to be addressed in the future. First, prospective long-term angiographic follow-up for patients with and without a TBS need to be conducted to determine whether a TBS develops simultaneously with the genesis of a BAVM or progressively develops after the formation of a BAVM. Second, laboratory investigations of local proangiogenic factors, such as basic fibroblast growth factor and/or vascular endothelial growth factor, in the dura mater or brain tissue of surgically treated patients were performed to determine the role of the BAVM-induced hypoxic environment in the formation of the TBS. Last, accurate and noninvasive measurement of the wall shear stress in the feeding arteries of the BAVM is needed to determine whether there are any differences with regard to wall shear stress between the patients with and without a TBS (4).

In this study, we found that there was no difference in age between the patients with and without a TBS, which was consistent with the study by Stein et al. (5). Of note, we found that patients with higher SM grades tended to have a TBS, which was also consistent with past reports (1, 3, 5).

### Classification of TBSs to BAVMs

To explore the heterogeneity of TBSs, Stein et al. classified TBSs into 3 types based on the proportion and intensity of BAVM nidi perfusion by ECA feeders (5). In the present study, we divided the TBSs into 3 types according to their sources as follows: TBSs originating from the unilateral ECA and/or VA, TBSs originating from the bilateral ECA and/or VA, and TBSs with involvement of the MHT (Figures 2, 4). According to this classification, we could determine the origin of the TBS, which would be helpful when performing EVT for BAVMs via the TBS. In addition, our classification system is also a supplement to that by Stein et al. (5).

### Location of a BAVM With a TBS

The occurrence of a TBS is affected by the location of the BAVM. As was reported, BAVMs located superficially in the subpial area had a TBS more frequently than deep-seated BAVMs (1, 3). According to Koo et al.'s report, BAVMs with a TBS are most commonly located in the temporal lobe, followed by the occipital and parietal lobes (3). Soderman et al. also reported that BAVMs with a TBS most commonly occurred in the temporal lobe, followed by the parietal lobe (1).

In accordance with previous reports, our study also showed that a TBS was more commonly noted in patients with temporal and parietal BAVMs. We speculated that the phenomenon of temporal lobe predilection might be occurring because the temporal dura is the major area supplied by the MMA and OA.

### Treatment and Prognosis of a BAVM With a TBS

The treatment of a BAVM with a TBS includes EVT, microsurgery, and stereotactic radiosurgery (2). Ideally, the goal of BAVM treatment is complete BAVM eradication. From this point of view, microsurgical resection (sometimes in combination with other treatments) is the modality of choice to achieve the highest rate of immediate and complete elimination of a BAVM nidus (13). In recent years, for ruptured BAVMs, the primary aim of EVT has been targeted embolization of the ruptured structures. For unruptured BAVMs, the primary target is embolization of the weak points. Therefore, EVT has increased in popularity (7).

In this study, 24 patients underwent targeted EVT. Six patients were managed conservatively, as no weak points or ruptured structures were identified (14). Flow-related aneurysms were identified in 5 patients, and they all underwent EVT. For ruptured BAVMs, if no aneurysm-like structure was identified in the nidus, the aim of EVT was to reduce blood volume in the area.

In addition to the treatment of the BAVM, the TBS was also considered. If the TBS was evidently enlarged, which indicated a high blood volume, endovascular embolization was performed. In this study, 10 patients underwent a TBS embolization.

In this case series, 26 (86.7%, 26/30) patients achieved good recovery, which indicated that EVT was also a reasonable option for this rare scenario. Two patients died during the follow-up period; the SM grades were 3 and 4 for these patients. However, they both had a type 1 TBS. Therefore, we speculate that a higher SM grade might have been a considerable contributor to rebleeding in the two patients who died (Figure 8).

## Limitation

This was a retrospective single-center study and only had a limited sample size. As a result of the economic status in rural areas in China, angiographic follow-up data could only be obtained in a small proportion of the patients in this series, which affected the interpretation of the angiographic outcomes.

## Conclusion

As a result of its rarity, there are still many questions regarding the angiographic and clinical characteristics that need to be answered for BAVMs with a TBS. This study showed that a TBS was likely to occur in patients with larger BAVMs and that a TBS was likely to be located in the temporal lobe in patients with BAVMs with higher SM grades. Also, the management protocol of BAVMs with a TBS was similar to that of BAVMs without a TBS. Weak structures were the primary targets of management. In addition, a BAVM could be embolized via the TBS.

## Data Availability Statement

The raw data supporting the conclusions of this article will be made available by the authors, without undue reservation.

## Ethics Statement

The studies involving human participants were reviewed and approved by the ethics committee of The First Hospital of Jilin University. Written informed consent for participation was not required for this study in accordance with the national legislation and the institutional requirements. Written informed consent was obtained from the individual(s) for the publication of any potentially identifiable images or data included in this article.

## Author Contributions

JY contributed to the conception and design of the manuscript. KH and KX wrote the manuscript. LQ and GL collected the medical records of the patients. JY and YG critically revised the manuscript. All authors approved the final version of this manuscript.

## Conflict of Interest

The authors declare that the research was conducted in the absence of any commercial or financial relationships that could be construed as a potential conflict of interest.

## References

[1] Soderman M Rodesch G Lasjaunias P. Transdural blood supply to cerebral arteriovenous malformations adjacent to the dura mater. AJNR Am J Neuroradiol. (2002) 23:1295–300. 12223368 PMC7976253

[2] Piao J Ji T Guo Y Xu K Yu J. Brain arteriovenous malformation with transdural blood supply: current status. Exp Ther Med. (2019) 18:2363–8. 10.3892/etm.2019.7731 31555346 PMC6755268

[3] Koo HW Jo KI Yeon JY Kim KH Jeon P Kim JS. Clinical features of superficially located brain arteriovenous malformations with transdural arterial communication. Cerebrovasc Dis. (2016) 41:204–10. 10.1159/000443530 26789929

[4] Bervini D Morgan MK Stoodley MA Heller GZ. Transdural arterial recruitment to brain arteriovenous malformation: clinical and management implications in a prospective cohort series. J Neurosurg. (2017) 127:51–8. 10.3171/2016.5.JNS16730 27588588

[5] Stein KP Moenninghoff C Kneist A Sandalcioglu IE Forsting M Sure U. Transdural blood supply in cerebral arteriovenous malformations: a Systematic evaluation of angioarchitecture. AJNR Am J Neuroradiol. (2018) 39:2307–12. 10.3174/ajnr.A5881 30409848 PMC7655402

[6] Chen CJ Ding D Derdeyn CP Lanzino G Friedlander RM Southerland AM. Brain arteriovenous malformations: a review of natural history, pathobiology, and interventions. Neurology. (2020) 95:917–27. 10.1212/WNL.0000000000010968 33004601

[7] Hou K Xu K Chen X Ji T Guo Y Yu J. Targeted endovascular treatment for ruptured brain arteriovenous malformations. Neurosurg Rev. (2020) 43:1509–18. 10.1007/s10143-019-01205-1 31720915

[8] Broderick JP Adeoye O Elm J. Evolution of the modified rankin scale and its use in future stroke trials. Stroke. (2017) 48:2007–12. 10.1161/STROKEAHA.117.017866 28626052 PMC5552200

[9] Yu J Guo Y Xu B Xu K. Clinical importance of the middle meningeal artery: a review of the literature. Int J Med Sci. (2016) 13:790–9. 10.7150/ijms.16489 27766029 PMC5069415

[10] Martins C Yasuda A Campero A Ulm AJ Tanriover N Rhoton A Jr. Microsurgical anatomy of the dural arteries. Neurosurgery. (2005) 56(2 Suppl):211-51. 10.1227/01.NEU.0000144823.94402.3D 15794820

[11] Hou K Guo Y Xu K Yu J. Clinical importance of the superficial temporal artery in neurovascular diseases: a PRISMA-compliant systematic review. Int J Med Sci. (2019) 16:1377–85. 10.7150/ijms.36698 31692910 PMC6818193

[12] Wang G Yu J Hou K Guo Y Yu J. Clinical importance of the posterior meningeal artery: a review of the literature. Neuroradiol J. (2019) 32:158–65. 10.1177/1971400919840843 30924401 PMC6512203

[13] van Beijnum J van der Worp HB Buis DR Al-Shahi Salman R Kappelle LJ Rinkel GJ. Treatment of brain arteriovenous malformations: a systematic review and meta-analysis. JAMA. (2011) 306:2011–9. 10.1001/jama.2011.1632 22068993

[14] Wong J Slomovic A Ibrahim G Radovanovic I Tymianski M. Microsurgery for ARUBA trial (A randomized trial of unruptured brain arteriovenous malformation)-eligible unruptured brain arteriovenous malformations. Stroke. (2017) 48:136–44. 10.1161/STROKEAHA.116.014660 27856955

